# Age Variations in Perceived COVID-19 Threats, Negative Impacts, and Associations with Well-Being

**DOI:** 10.1093/geroni/igaa057.3420

**Published:** 2020-12-16

**Authors:** Tara Gruenewald, Anthony Ong, Danielle Zahn

**Affiliations:** 1 Chapman University, Orange, California, United States; 2 Cornell University, Ithaca, New York, United States

## Abstract

The COVID-19 pandemic represents an unprecedented threat to individual and public health, psychosocial, and economic well-being, although COVID-19 threats and impacts may vary by age and other demographic characteristics. Although greater age is a risk factor for greater COVID-19 disease severity, we know little about the association between age and perceived and experienced COVID-19 threats and their association to well-being. These associations were examined in an ongoing 3-wave investigation of over 1,700 U.S. adults (age 18-89; 53.1% female). Wave 1 analyses indicate no significant age variation in perceived threat of COVID-19 infection, with older and younger individuals reporting similar levels of COVID-19 infection threat. However, greater age was associated with lower perceived negative impact on financial and needed resources (r=-.10**), lower perceptions of COVID-19 induced harm to mental well-being (r=-.17**), and more favorable well-being profiles. Greater perceived COVID-19 threat and negative impact on resources and well-being were linked to greater feelings of stress (β’s=.45 to .68***), loneliness (β’s=.24 to .49***), social well-being (β’s=-.19 to -.36***), and poor sleep quality (β’s=.34 to .51***). These associations did not vary with age with the exception that older individuals showed stronger links between COVID-19 threat and impacts and poorer sleep quality. Ongoing analyses are examining whether these associations persist over time. Despite older adults’ greater risk of COVID-19 disease severity and mortality, older age did not appear to be linked to greater perceived COVID-19 threat or impacts, nor linkages to ill-being, with the possible exception of potential greater vulnerability to poor sleep quality.

